# Genetic diversity among the present Japanese population: evidence from genotyping of human cell lines established in Japan

**DOI:** 10.1007/s13577-024-01055-0

**Published:** 2024-04-19

**Authors:** Fumio Kasai, Makoto Fukushima, Yohei Miyagi, Yukio Nakamura

**Affiliations:** 1 Cell Engineering Division, BioResource Research Center, RIKEN Cell Bank, Tsukuba, Japan; 2 Molecular Pathology and Genetics Division, Kanagawa Cancer Center Research Institute, Yokohama, Japan

**Keywords:** Asian population, Cellular resource, Genetic admixture, Japanese genome, Lung cancer

## Abstract

Japan is often assumed to have a highly homogeneous ethnic population, because it is an island country. This is evident in human cell lines collected from cell banks; however, these genotypes have not been thoroughly characterized. To examine the population genotypes of human cell lines established in Japan, we conducted SNP genotyping on 57 noncancerous cell lines and 43 lung cancer cell lines. Analysis of biogeographic ancestry revealed that 58 cell lines had non-admixed Japanese genotypes, 21 cell lines had an admixture of Japanese and East Asian genotypes, and the remaining 21 cell lines had East Asian genotypes. The proportion of non-admixed Japanese genotypes was similar between lung cancer and noncancerous cell lines, suggesting that patients in Japan may not exclusively have Japanese genotypes. This could influence the incidence of inherited diseases and should be taken into account in personalized medicine tailored to genetic background. The genetic makeup of the present-day Japanese population cannot be fully explained by the ancestral Jomon and Yayoi lineages. Instead, it is necessary to consider a certain level of genetic admixture between Japanese and neighboring Asian populations. Our study revealed genetic variation among human cell lines derived from Japanese individuals, reflecting the diversity present within the Japanese population.

**Supplementary Information:**

The online version contains supplementary material available at 10.1007/s13577-024-01055-0.

## Introduction

Japan is located at the eastern end of Asia and is composed of more than 14,000 islands, including the four main islands. The population history of Japanese people is influenced by geographic factors, which can be explained by the dual structure model based on morphological characteristics [1]. The model illustrates two lineages: the Jomon lineage, which originated from Southeast Asians, followed by the Yayoi lineage, which originated from Northeast Asians. A large number of immigrants were brought into Japan from the Korean Peninsula after the Yayoi period [2]. Genetic distance analysis revealed that the Ainu and Ryukyuan people are descendants of the Jomon people, whereas the Hondo-Japanese population is closely related to Koreans, reflecting the Yayoi lineage [3]. An analysis of ancient populations proposed that modern Japanese people are the result of admixture between Jomon hunters and Yayoi farmers [4]. It has been suggested that the Yayoi genome was gradually diluted through repeated admixture processes [5]. Analysis of the present Japanese genotypes at the prefecture level suggested that genetic heterogeneity in mainland Japan was caused by admixture between the Jomon and Yayoi ancestors [6]. Whole-genome sequencing explored the evolutionary processes behind the genetic diversity of the Japanese population [7]. These genetic studies demonstrated the formation of modern Japanese populations, indicating that Japanese genomes can be distinguished from those of other Asian ethnic groups.

In contrast to anthropological studies of Japanese ancestry, genomic medicine considers the ethnic background of patients. It has been reported that variations in genotypes among population groups could be linked to differences in disease incidence or drug sensitivity [8]. Ethnic disparities have been documented in both the incidence of lung cancer and the efficacy of therapeutic treatments [9, 10]. Sequence variant analysis of lung cancer revealed differences in mutation profiles between Japanese and Caucasian populations [11]. This suggests that genetic background, in addition to environmental factors, influences the development of lung cancer. Prospective cohort studies have been conducted to identify genomic signatures associated with disease in the Japanese population [12]. The Japanese genome sequence has been assembled to serve as a population-specific reference, with the aim of facilitating precision medicine [13]. However, the majority of widely used cancer cell lines are of European ancestry [14], resulting in limited variation in cellular models.

Although genetic information from the Japanese population has accumulated in databases, reference materials corresponding to genomic data are often unavailable. Human cell lines serve as in vitro cellular models in various fields, including for the investigation of pathological mechanisms and drug development. The RIKEN Cell Bank has a vast collection of cellular resources that were primarily established in Japan; however, their population genotypes have not been assessed. Previous studies that have investigated the genetic ancestral information of human cell lines have mainly focused on cancer cell lines [14–16]. Because tumor cells often have abnormal genomes, it is necessary to use cells derived from normal tissues as reference controls. In this study, we performed population genotyping on both lung cancer and noncancerous cell lines established in Japan.

## Materials and methods

### Cell lines and DNA preparations

In this study, 100 cell lines registered with the RIKEN Cell Bank (RCB) were selected, comprising 43 lung cancer cell lines, 32 Epstein-Barr Virus transformed lymphoblastoid cell lines (LCLs) and 25 cell lines established from noncancerous tissues (Table S1). The LCLs included a collection established from the peripheral blood mononuclear cells (PBMCs) of healthy volunteers in Japan [17] and were designated as HEVs (Human Epstein-Barr Virus transformed cells). The lung cancer cell lines originated from 10 adenocarcinomas, 9 large cell carcinomas, 8 squamous cell carcinomas, and 16 small cell carcinomas. These cell lines were all established by laboratories in Japan, assuming that patients and donors would be residents of Japan. Genomic DNA was extracted using the AllPrep DNA/RNA Mini Kit (Qiagen, 80204).

### Sequence analysis

An ancestry-informative SNP analysis was conducted using the Ion AmpliSeq Precision ID Ancestry Panel (A25642), which targets 165 SNPs. Sequence libraries and templates were prepared using the Ion AmpliSeq Kit for Chef DL8 (Thermo Fisher Scientific, A29024), followed by the Ion 510^™^ & Ion 520^™^ & Ion 530^™^ Kit–Chef (Thermo Fisher Scientific, A34461). Sequencing was performed on the Ion GeneStudio S5 System using the Ion 520 chip (Thermo Fisher Scientific, A27763). The reads were aligned to the hg19 reference. The population likelihoods were estimated based on the clustering of 66 population groups using the HID SNP Genotyper plugin v5.2.2 (Thermo Fisher Scientific).

## Results

SNP genotyping of 100 cell lines identified population groups from East Asia, as listed in Table 1. The likelihood values are shown in supplementary Table 2. Some samples have the first two likelihood values in close proximity, indicating that it would not be adequate to classify them as a single population. It is assumed that these genomes could be composed of an admixture of two populations. In this study, genomes were classified into two groups: non-admixed and admixed genotypes. Non-admixed genomes are those classified as a single group, while admixed genomes are classified into two groups.

**Table 1:** Genotypes of 100 human cell lines

A. List of 58 cell lines classified into Japanese genotypes			
Cell type	Cell name	Cell type	Cell name
Normal	HE31	LCL	HEV0208
	HFL-AE-III		HEV0236
	HFL-I		HEV0295
	HFSK9t		HEV0300
	HFSKF-AE-V		HEV0333
	HS-K		HEV0388
	HUC-F		HEV0410
	HUC-F2	LC	86-2
	HUC-Fm		A110L
	HUC-Fm2		A529L
	NB1RGB		HLC-1
	NHSF46		IA-LM
	SF8406		LC-1 sq
	TIG-1		LC-2 ad
	TIG-7		LK-2
	UCB-TERT-21		Lu-24
LCL	CB-3512		Lu-134-A
	HEV0011		Lu-135
	HEV0024		Lu-139
	HEV0031		Lu-143
	HEV0032		LU65
	HEV0037		MCC138c
	HEV0039		MS-1
	HEV0054		RERF-LC-KJ
	HEV0098		S1
	HEV0101		Sq-1
	HEV0114		T3M-11
	HEV0121		WA-hT
	HEV0149		Y-ML-1B

B. List of 21 cell lines classified into Japanese admixed genotypes		
Genotype	Cell name	Cell type
Japanese-Korean	HFL-II	Normal
	TIG-3	
	UE6E7-16	
	HEV0012	LCL
	HEV0178	
	HEV0240	
	HEV0251	
	HEV0380	
	HEV0421	
	87-5	LC
	Lu99	
Japanese-Hakka	HEV0034	LCL
	HEV0404	
	HEV0500	
	B901L	LC
	Lu-140	
Japanese-Han	HEV0218	LCL
	HEV0498	
	PC-9	LC
Japanese-Lao	A129L	LC
Japanese-Micro	II-18	LC

C. List of 21 cell lines classified into Easi Asian genotypes		
Genotype	Cell name	Cell type
Korean	HE40	Normal
	HFSKF-II	
	HFL-AE-VI	
	HEV0325	LCL
	C831L	LC
	LCAM1	
	Lu-141	
	MCC148c	
Hakka	HE16	Normal
	HFL-III	
	HFL-AE-VII	
	T3M-12	LC
Taiwanese	EBC-1	LC
	RERF-LC-AI	
	T3M-10	
Han	G603L	LC
Lao	S2	LC
Han-Korean	A904L	LC
Han-Taiwanese	Lu-138	LC
Lao-Han	B1203L	LC
Hakka-Taiwanese	Lu-165	LC

*LCL* lymphoblastoid cell line, *LC* lung cancer

**Fig. 1:**
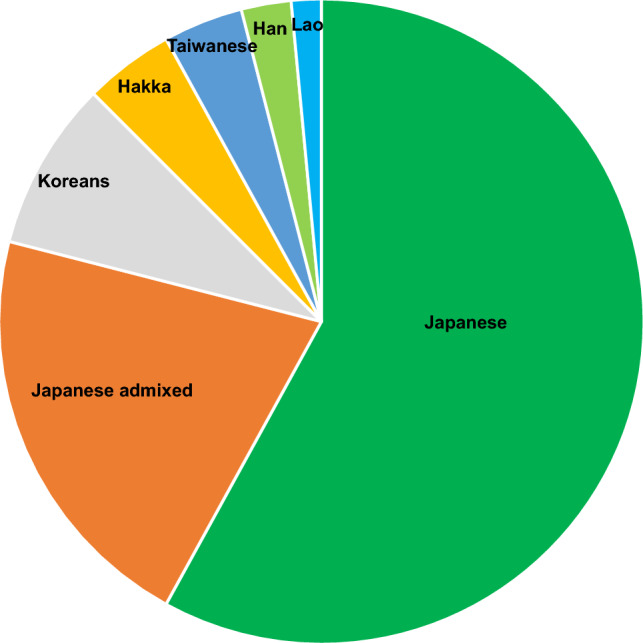
Distribution of population groups from SNP genotyping of a total of 100 human cell lines. Japanese genotypes, including East Asian admixtures, accounted for 81% of the total. Admixed genotypes between Japanese and East Asian individuals constituted 35% of the 81 cell lines. The remaining 19 cell lines were of East Asian origin. Among them, six cell lines were detected as admixed genotypes. These were counted as half of the non-admixed genotypes and classified into five subpopulations in this graph

Among the 100 genomes, 58 cell lines were identified as having non-admixed Japanese genotypes, while 21 cell lines were identified as having admixed Japanese genotypes (Fig. 1). The remaining 21 cell lines were identified as having East Asian genotypes that are different from those of Japanese individuals. These included seven Korean, three Hakka, three Taiwanese, one Han, and one Lao non-admixed genotype. Additionally, there are four admixed genotypes that are admixtures of two non-Japanese East Asian genotypes. ‘Han’ refers to the ethnic group of Han Chinese, which makes up 92% of the Chinese population [18]. Hakka is a subpopulation of Han Chinese with roots in Guangdong, South China. ‘Lao Loum’ refers to the people who are indigenous to the lowland regions of Laos.

Two datasets for Japanese SNP data, 1000 Genomes and HapMap, were used for analyzing ancestry genotyping with the HID SNP Genotyper. Among 58 non-admixed Japanese cell lines, 36 and 14 cell lines were also predicted by the other Japanese dataset to have the second and third highest likelihood estimates, respectively (Table S2). This resulted in a total of 50 cell lines being predicted by both datasets within the top three likelihood estimates. In contrast, the two datasets are present in 13 out of 21 admixed Japanese and 3 out of 21 non-Japanese cell lines among the three highest likelihood estimates. Six out of 21 non-Japanese cell lines were not identified by the two Japanese datasets in the top three highest likelihood estimates.

**Fig. 2:**
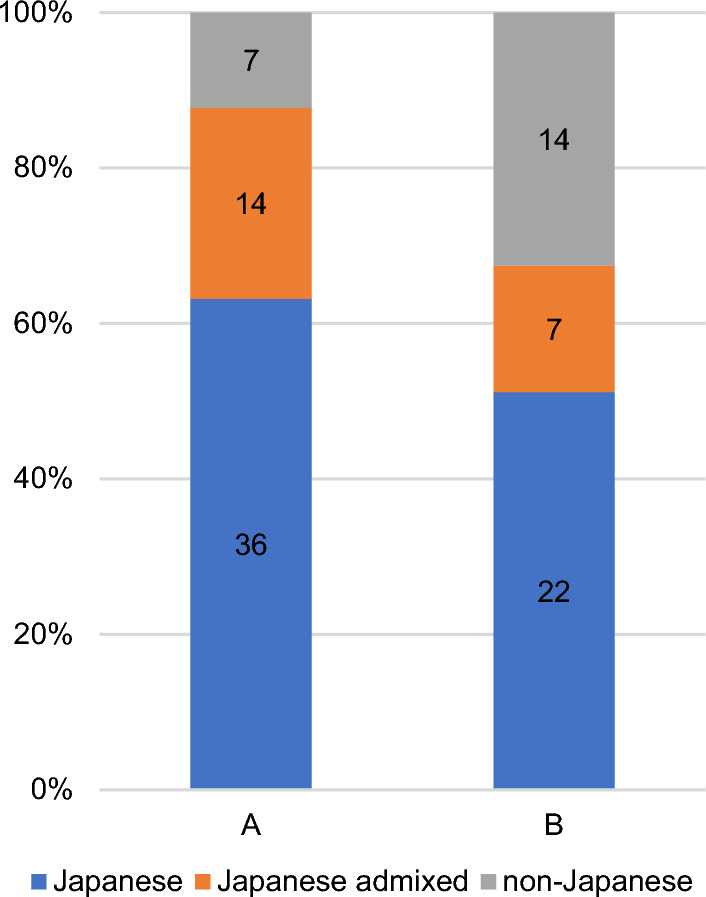
Comparison of genotypes between noncancerous (**A**) and tumor (**B**) samples based on analysis of 57 noncancerous and 43 lung cancer cell lines. The proportions of non-admixed Japanese genotypes were similar between the two cell types. Differences are observed in admixed Japanese genotypes. Among the non-Japanese genotypes, seven non-admixed and six admixed genotypes were detected in the lung cancer cell lines, while no admixed genotypes were detected in the noncancerous cell lines. This difference could be caused by genomic alterations in tumor cells, resulting in changes in genotypes. The numbers in the graphs indicate the number of cell lines

When comparing noncancerous cell lines to lung cancer cell lines, non-admixed Japanese genotypes accounted for 65% and 54%, respectively (Fig. 2). Japanese genomes, including admixed genotypes, make up 90% of the noncancerous cell lines and 70% of the lung cancer cell lines. Among the HEV cell lines, 61% and 36% are Japanese non-admixed and admixed genotypes, respectively, with the exception of one Korean non-admixed genotype out of 31 cell lines (Figure S1). This is partly because the HEV cell line was designed to establish cell lines from Japanese individuals.

## Discussion

The Japanese archipelago is not connected to the Eurasian continent, leading to the isolation of the Japanese population. It is assumed that few admixture events occurred in Japan after the early migratory waves of the Jomon and Yayoi periods, unlike on continents where populations are admixed [19]. In contrast, our results demonstrate that approximately 60% of human cell lines have genomes with typical Japanese genotypes. Additionally, 20% of them have genomes that are a mixture of Japanese and East Asian ancestry, while the remaining 20% have genotypes of East Asian origin other than Japanese. This experimental evidence indicates diversity within the Japanese population and genomes, challenging the conventional view that the present-day Japanese population is largely mono-ethnic. It is suggested that recent population admixture in East Asian populations lead to increased population genetic diversity [20]. Our results imply that genetic admixture should be taken into account when analyzing Japanese genomes and delivering genomic medicine.

Cultural and social practices could influence genetic variation among ancient populations [21]; however, these factors would not serve as barriers between the present populations. The origin of East Asians has little relevance to the genetic diversity of populations in East Asia [22]. Analysis of SNP genotypes among Japanese individuals revealed that a small fraction of individuals were not classified within the Japanese cluster, suggesting that some of these individuals have genetic backgrounds that are not solely Japanese but rather a mixture of Japanese and non-Japanese East Asians [23]. Admixture from surrounding groups serves as the principal driving force that causes and maintains genetic diversity, because it can rapidly alter the gene pool in a single generation and introduce new genetic materials for adaptation [24]. It is expected that recent population admixture in East Asia has increased genetic diversity [25], implying that the current population of Japan cannot be solely explained by Japanese genotypes.

A number of publications have reported the genomes of cancer clinical cases in Japan, some of which are based on the assumption that Japanese patients have Japanese genotypes. According to census data as of December 2022, foreign residents in Japan account for 2.5% of the population (Table S3-1). Notably, 84.4% of the respondents came from Asian countries. According to statistics from 2013, 3.1% of all live births were from at least one foreign parent, including 1.9% of births from parents where one was Japanese and the other was a foreigner (Table S3-2). Genotype and nationality are not always correlated [21], even though Japan is geographically isolated as an island nation. The ratio of Japanese genotypes was similar between lung cancer and noncancerous cell lines in this study. This suggests that cell lines established from Japanese patients or donors may not necessarily have genomes derived solely from individuals of Japanese ancestry, indicating that patients in Japan exhibit genetic diversity and belong to the East Asian population group.

The cell lines analyzed in this study were derived from 60 males and 30 females (Table S1, Figure S2). The gender distribution of the lung cancer cell lines included 36 males and 5 females. Clinical materials used to establish lung cancer cell lines are often derived from patients in advanced stages of lung cancer. Among them, males account for more than 70% [26], which likely explains the heavily biased distribution toward men.

Samples used in this study were not prepared exclusively for this analysis but had already been deposited as a collection of cellular resources. Due to this limitation, along with a small sample size, sampling bias is inevitable, and patients in metropolitan areas would include various ethnicities besides Japanese. In fact, this study only identified foreign genotypes from East Asia, and cases from Europeans, Americans, or Africans were not included. Even though 60% of the cell lines in this study are classified as non-admixed Japanese ancestry, the likelihood values vary among those samples. Our study may not accurately reflect the ethnic genotypes in Japan; however, variations in genotypes among cell lines established from Japanese residents indicate the presence of a certain degree of admixed genotypes within the Japanese population.

In this study, 25 out of 100 cell lines were detected as admixed genotypes, assuming that these individuals are second-generation offspring of parents belonging to different population groups. Due to the complexity, mixed genotypes beyond the third generation cannot be discriminated, resulting in the detection of the dominant genotype within the genome as non-admixed. This suggests that individuals identified as having non-admixed Japanese genotypes could have ancestry belonging to East Asians other than Japanese. Unlike intergenerational changes, tumor genomes can undergo alterations during proliferation, which can result in abnormal genomes containing mutations, gains, and/or losses [27]. These alterations can affect SNP patterns in tumor genomes, and extensive loss of heterozygosity at the chromosome level affects genotype shifting from admixed to non-admixed [28]. Although two lung cancer cell lines, C831L and MCC148cc, are classified as Korean, they are distinct among the non-Japanese group, because they are predicted as Japanese by two datasets with the second and third highest likelihood estimates. This would be reflected by extensive genomic alterations in tumor cells. As the proportion of admixed genomes in cancer cell lines is lower than that in noncancerous cell lines, non-admixed genotypes would have been derived from admixed genomes. Although this approach may have limitations in terms of target regions, it is still possible to assess differences or similarities between samples through this analysis. Further analysis may provide insight into the genome composition of admixed populations and will reassess the classification of population groups.

Analysis of Asian populations using the Precision ID Ancestry panel demonstrated that Chinese, Japanese, and Korean individuals were correctly classified as East Asian, showing the highest likelihood values for ethnicity based on reference data [29]. Although the panel did not perform well in assigning subpopulations for self-declared Oceanian and American individuals, the subpopulation prediction was accurate for the majority of self-declared East Asian individuals [30]. These previous studies, which used the same method, support our approach, implying that our results can serve as primary screening data. It has been reported that a panel of 142 SNP markers can distinguish Japanese people from Chinese Han people and Koreans with an overall average accuracy of more than 90% [31]. This indicates that an alternative analysis that focuses on East Asian ancestry-informative SNPs may refine our results.

It is reported that 88% of early established cell lines, which are commonly used worldwide, established in USA, suggesting a lack of ancestral diversity [32]. This indicates that several studies, including those conducted in Japan, have utilized human cell lines with non-Japanese genotypes, such as HeLa, A549, K-562, and others. Cell lines are not necessarily limited to non-admixed Japanese genotypes, and the unique characteristics of cell lines can serve as cellular models regardless of genetic admixtures. Given that Japanese genomes are unlikely to have remained highly conserved throughout human evolution, cell lines with mixed genotypes could serve as a model for understanding the evolution of East Asian populations. Analysis of genetic ancestries has been conducted; however, DNA samples are not always publicly available, and the samples used in the previous studies cannot be reused for subsequent research. Compared with limited genomic DNA samples obtained from small amounts of specimens, such as PBMCs, human cell lines characterized by population groups have an advantage in terms of sharing genetic resources between laboratories.

Variations in population genotypes among human cell lines established in Japan would be reflected in increasing immigration from neighboring Asian countries as internationalization expands beyond geographical barriers. Differences in population groups among cell lines result in cellular materials with variable genetic backgrounds, and this factor needs to be considered in the analysis. We demonstrate the genetic diversity of human cell lines and suggest that in vitro experiments should utilize a variety of cellular resources rather than solely focusing on representative cell lines.

### Supplementary Information

Below is the link to the electronic supplementary material.

Supplementary file1 (PDF 149 KB)

Supplementary file2 (XLSX 18 KB)

## Data Availability

The data that support the findings of this study are available from the corresponding author upon request.
