# Supplementary material for: Genetic diversity among the present Japanese population: evidence from genotyping of human cell lines established in Japan
Source: Hum Cell. 2024 Apr 19;37(4):944–50. doi: 10.1007/s13577-024-01055-0 (PMC11194210; doi:10.1007/s13577-024-01055-0)
Supplement: Supplementary file 1 — Supplementary file1 (PDF 149 KB) [file 13577_2024_1055_MOESM1_ESM.pdf]

Table S1. List of 100 cell lines.

Classification	Tissue/Histology		Cell Name	Cell No	Sex	Age
Normal	Lung		HFL-I	RCB0521	M	Fetus
			HFL-II	RCB0522	M	Fetus
			HFL-III	RCB0523	M	Fetus
			HFL-AE-III	RCB0684	UN	15FW
			HFL-AE-VI	RCB1613	UN	Fetus
			HFL-AE-VII	RCB1617	UN	Fetus
			TIG-1	RCB4467	F	0
			TIG-3	RCB4468	M	0
			TIG-7	RCB4469	M	0
	Skin		HFSK9t	RCB1542	M	15FW
			HFSKF-II	RCB0698	F	Fetus
			HFSKF-AE-V	RCB1139	UN	Fetus
			HS-K	RCB0159	F	UN
			NB1RGB	RCB0222	M	0
			NHSF46	RCB0162	M	46
			SF8406	RCB0584	F	14
	Umbilical cord		HUC-F	RCB0153	F	0
			HUC-F2	RCB0436	F	0
			HUC-Fm	RCB0197	M	0
			HUC-Fm2	RCB0437	M	0
	Embryo		HE16	RCB2237	UN	0
			HE31	RCB2248	UN	0
			HE40	RCB2259	UN	0
	MSC	BM	UE6E7-16	RCB2163	F	91
		UC	UCB-TERT-21	RCB2079	M	0
	Lymphoblastoid		CB-3512	RCB2531	UN	0
			HEV0011	HEV0011	M	27
			HEV0012	HEV0012	F	46
			HEV0024	HEV0024	F	39
			HEV0031	HEV0031	F	65
			HEV0032	HEV0032	M	64
			HEV0034	HEV0034	M	31
			HEV0037	HEV0037	M	54
			HEV0039	HEV0039	M	65
			HEV0054	HEV0054	F	47
			HEV0098	HEV0098	M	36
			HEV0101	HEV0101	M	57
			HEV0114	HEV0114	F	56
			HEV0121	HEV0121	F	37
			HEV0149	HEV0149	F	39
			HEV0178	HEV0178	F	39
			HEV0208	HEV0208	F	30
			HEV0218	HEV0218	M	46
			HEV0236	HEV0236	F	34
			HEV0240	HEV0240	F	39
			HEV0251	HEV0251	M	67
			HEV0295	HEV0295	M	71
			HEV0300	HEV0300	F	34
			HEV0325	HEV0325	M	54
			HEV0333	HEV0333	F	29
			HEV0380	HEV0380	F	24
			HEV0388	HEV0388	F	52
			HEV0404	HEV0404	M	72
			HEV0410	HEV0410	F	57
			HEV0421	HEV0421	M	70
			HEV0498	HEV0498	F	26
			HEV0500	HEV0500	F	28

Classification	Tissue/Histology	Cell Name	Cell No	Sex	Age
Lung cancer	Adenocarcinoma	A110L	RCB2816	M	61
		A129L	RCB3532	M	64
		A529L	RCB2817	M	72
		B901L	RCB3530	F	72
		HLC-1	RCB0083	UN	UN
		II-18	RCB2093	UN	UN
		LC-2 ad	RCB0440	F	51
		LCAM1	RCB1425	M	53
		PC-9	RCB4455	M	45
		RERF-LC-KJ	RCB1313	M	78
	Large cell carcinoma	86-2	RCB2134	M	36
		A904L	RCB3531	M	46
		C831L	RCB2819	M	54
		G603L	RCB3529	M	75
		IA-LM	RCB0554	M	68
		LU65	RCB1967	M	64
		Lu99	RCB1900	M	63
		T3M-10	RCB1020	M	40
		Y-ML-1B	RCB5394	M	70
	Squamous cell carcinoma	B1203L	RCB2818	M	52
		EBC-1	RCB1965	M	69
		LC-1 sq	RCB0455	M	68
		LK-2	RCB1970	M	74
		MCC138c	RCB5007	M	75
		MCC148c	RCB5008	M	84
		RERF-LC-AI	RCB0444	M	UN
		Sq-1	RCB1905	M	UN
	Small cell carcinoma	87-5	RCB2092	M	64
		Lu-134-A	RCB0466	M	80
		Lu-135	RCB0468	M	69
		Lu-138	RCB1785	M	78
		Lu-139	RCB0469	M	63
		Lu-140	RCB0470	M	53
		Lu-141	RCB1772	F	67
		Lu-143	RCB1773	M	60
		Lu-165	RCB1184	M	50
		Lu-24	RCB1771	F	42
		MS-1	RCB0725	M	54
		S1	RCB1966	F	58
		S2	RCB2133	M	UN
		T3M-11	RCB1022	M	UN
		T3M-12	RCB2281	M	UN
		WA-hT	RCB2279	M	56

UN: unspecified

Table S3.

S3-1. Population statistics as of December 2022.

Total population estimate	12484×10^4
Foreign residents	3,075,213
Asian	2,594,216

Data obtained from the government statistics portal (e-Stat).

S3-2. Vital Statistics as of 2013.

Total live births	1,042,813
Both or one foreign parent	32,529
Japanese and foreign parents	19,532

Data obtained from the government statistics portal (e-Stat).

Figure S1

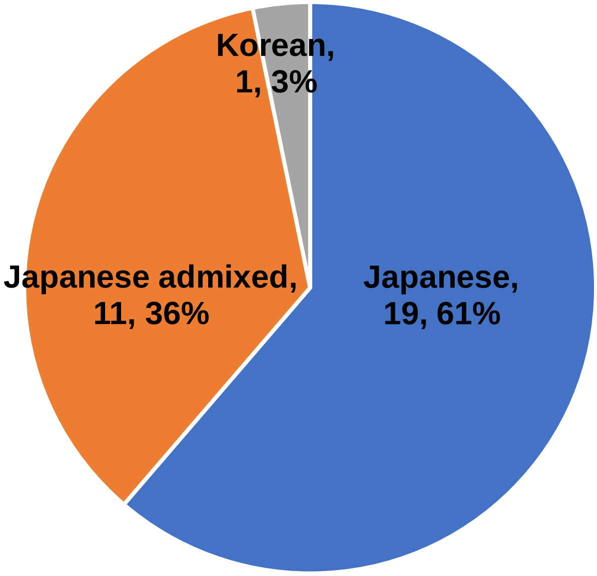

Distribution of population groups focused on 31 HEV cell lines. Japanese genotypes, including East Asian admixture, accounted for 97%, with the exception of one Korean genotype. Numbers in the graphs indicate the number of cell lines.

Figure S2

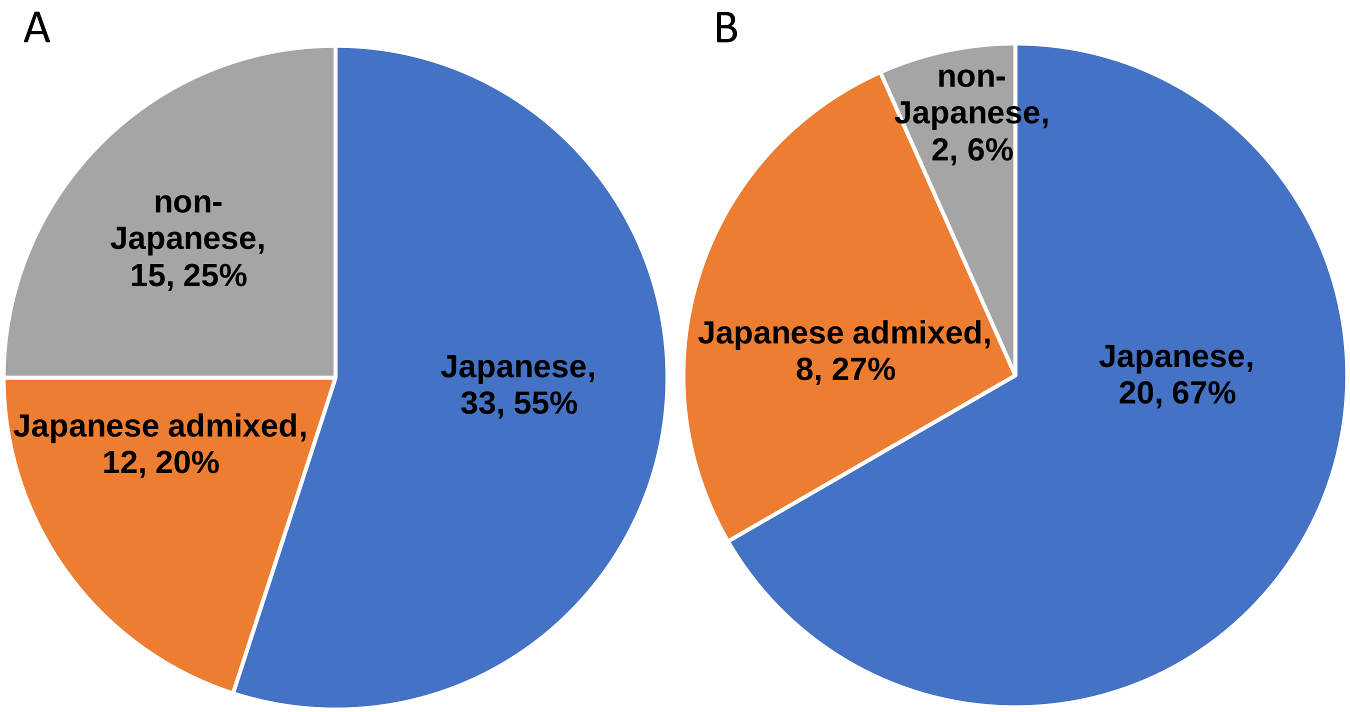

Comparison of genotypes between male (A) and female (B) cell lines. These data are based on samples of 60 males and 30 females. Differences in sample size are influenced by bias resulting from a higher number of male lung cancer patients. Numbers in the graphs indicate the number of cell lines.
